# Sarcopenic Obesity in Children and Adolescents: A Systematic Review

**DOI:** 10.3389/fendo.2022.914740

**Published:** 2022-06-01

**Authors:** Marcela Zembura, Paweł Matusik

**Affiliations:** Department of Pediatrics, Pediatric Obesity and Metabolic Bone Diseases, Chair of Pediatrics and Pediatric Endocrinology, Faculty of Medical Sciences in Katowice, Medical University of Silesia, Katowice, Poland

**Keywords:** sarcopenia, obesity, muscle mass, muscle strength, children, adolescents

## Abstract

Sarcopenic obesity (SO) is defined as co-occurrence of increased fat mass and sarcopenia and may predict adverse health outcomes in the pediatric population. However, the prevalence of SO and its association with adverse health outcomes have not been well defined in children and adolescents. We systematically reviewed data on the SO definition, prevalence, and adverse outcomes in the pediatric population. A total of 18 articles retrieved from PubMed or Web of Science databases were included. Overall, there was a wide heterogeneity in the methods and thresholds used to define SO. The prevalence of SO ranged from 5.66% to 69.7% in girls, with a range between 7.2% and 81.3% in boys. Of the 8 studies that evaluated outcomes related to SO, all showed a significant association of SO with cardiometabolic outcomes, non-alcoholic fatty liver disease (NAFLD) severity, inflammation, and mental health. In conclusion, this review found that SO is highly prevalent in children and adolescents and is associated with various adverse health outcomes. Findings of this review highlight the need for the development of a consensus regarding definition, standardized evaluation methods, and age and gender thresholds for SO for different ethnicities in the pediatric population. Further studies are needed to understand the relationship between obesity and sarcopenia and SO impact on adverse health outcomes in children and adolescents.

## 1 Introduction

According to the World Health Organization (WHO), over 340 million children and adolescents aged 5–19 years were overweight or obese in 2016 (1). Despite the implementation of numerous obesity prevention programs, the prevalence of overweight and obesity among children and adolescents is rising on each of the continents. The growing prevalence of childhood obesity is associated with noncommunicable comorbidities affecting almost every system in the body, including insulin resistance (IR), type 2 diabetes mellitus (T2DM) (2), elevated blood pressure (3), dyslipidemia (4), non-alcoholic fatty liver disease (NAFLD) (5), obstructive sleep apnea (OSA) (6), and psychosocial sphere (7).

The term “sarcopenia” (Greek “sarx” or flesh + “penia” or loss) was first introduced in 1989 by Irwin Rosenberg as the term to describe the decrease of muscle mass in the population of the elderly (8, 9). Since then, major changes occurred in the definition of sarcopenia and led to the development of The European Working Group on Sarcopenia in Older People (EWGSOP), which proposed a new definition of *sarcopenia* involving the assessment of muscle function. The EWGSOP recommended using the presence of both low muscle mass (LMM) and low muscle function (strength or performance) for the diagnosis of sarcopenia (10). However, according to EWGSOP2 revised consensus from 2019, muscle strength is now the primary parameter of sarcopenia. The occurrence of sarcopenia is probable when low muscle strength is observed. To confirm sarcopenia diagnosis, low muscle quantity or quality must be detected. When low muscle strength, low muscle quantity/quality, and low physical performance co-occur, sarcopenia is regarded as severe (11).

“Sarcopenic obesity (SO) is an emerging clinical entity characterized by excessive fat mass in the presence of reduced muscle mass (12)”. The co-occurrence of sarcopenia and obesity indicates a synergistically amplified risk of adverse health outcomes (13).

Sarcopenia and SO were once considered as afflictions affecting only the elderly, regarding the changes in body composition with aging involving muscle mass decline after the fourth decade (14), reduced resting metabolic rates (15), and reduced metabolic adaptation (16). A reduction in energy expenditure is not in line with a decrease in appetite that can lead to the development of obesity (16). Correlation of this factors leads to the development of SO. Furthermore, the sedentary lifestyle of elderly people further exacerbates changes in metabolism and body composition (16).

However, sarcopenia is now also linked to the pediatric population, as sarcopenia was found to be a risk factor of insulin resistance and higher metabolic risk in children and adolescents (17, 18). There is no established consensus regarding SO definition, diagnostic methods, and age- and gender-specific cutoff points in children and adolescents.

Currently, a wide range of various techniques are used to estimate muscle mass. Computed tomography (CT) and magnetic resonance imaging (MRI) are considered gold standards for estimating muscle mass because of their accuracy (10). However, few factors limit the usage of imaging techniques for routine clinical practice, including high cost, limited access to equipment, radiation exposure, and contraindications for scanning (19). The use of gold standard methods in the pediatric population has been limited mostly to patients with end-organ failure and children with solid/hematologic malignancies. Moreover, there is no consensus regarding landmarks (L2–L5) and muscle type/number used in studies concerning the pediatric population; most commonly used are the measures of psoas muscle area or psoas muscle index measured at L3 on CT, although some studies used L2, L4, or L5; MRI; paraspinous muscle area; or intramuscular adipose tissue area (20). Dual-energy X-ray absorptiometry (DXA) is now considered to be the most widely utilized method for muscle mass quantification because this method is quick, simple, low-cost, more available than CT/MRI, and is associated with minimal radiation exposure (10, 19, 21). The usage of bioelectrical impedance analysis (BIA) is inexpensive, readily reproducible, and appropriate for both ambulatory and bedridden patients (10). It has been found that BIA results under standard conditions correlate well with MRI predictions (22). However, susceptibility to patient hydration status is considered to be the main disadvantage of both DXA and BIA (20). Nevertheless, usage of these methods may be clinically warranted to assess for sarcopenia when CT and MRI are unavailable (23). Considering the fact that muscle mass is influenced by body size, absolute skeletal muscle (SM) mass (SMM) indicators should be adjusted for body size using height squared [appendicular skeletal muscle mass (ASM)/height^2^], weight (ASM/weight), or body mass index (BMI) (ASM/BMI) (24). Both total/partial body potassium per fat-free soft tissue and anthropometric measurements are not routinely used in clinical practice (10). There are a few studies providing reference values for DXA- and BIA-derived muscle mass parameters in children (25–28). Whereas only one study warranted reference values regarding CT-derived parameters of muscle mass in children (29). Assessment of muscle function should always be performed, since muscle strength is not linearly related to muscle mass (30). In older children and adolescents, strength and performance tests utilized in adults such as handgrip test, chair stand test, Timed Up and Go test, 6-min walk test, stair climb power test, and 400-m walk test can be used (10, 11). For younger children, standardized motor function assessment scales can be used in order to evaluate motor performance that may be affected by impaired muscle function. For this purpose, the validated Alberta Infant Motor Scale and the Peabody Developmental Motor Scale can be used (31).

To our knowledge, until now, no systematic reviews regarding solely SO in children and adolescents have been published. In this review, we aim to assess the current state of knowledge about SO among children and adolescents and its prevalence and evaluate outcomes related to SO, answering the question: “What are the diagnostic criteria, prevalence, and outcomes related to sarcopenic obesity in the population of children and adolescents?” in accordance to “participants, interventions, comparisons, and outcomes (PICO)” scheme.

## 2 Materials and Methods

A literature search of articles published in English was completed *via* PubMed and Web of Science databases on October 25, 2021. The following search query was used: (pediatrics or pediatric or childhood or child or children or adolescent or adolescence) AND (obese or obesity or overweight or adiposity or excessive weight) AND (sarcopenia or sarcopenic or muscle loss or sarcopenic obesity). Adequate filter regarding publication language (English) was applied. The search was done without limiting the years of publication. Two independent researchers (MZ, PM) screened the results. The inclusion criteria were studies that assessed the concept of SO among the pediatric population (<21 years) (32). Studies that addressed outcomes related to SO were also included. Studies in which the age of the control group exceeded age criterium and studies that evaluated only one sex were ruled out. Articles were excluded if they were editorials, letters, replies from authors, review articles, commentaries, case reports, articles conducted on animal models or cell culture, non-English articles, and articles without a full text. Studies performed in a population of children with neuromuscular disorders (e.g., Duchenne muscular dystrophy, spinal muscular atrophy) and autoimmune diseases affecting SM (e.g., dermatomyosis) were excluded considering possible disease impact on body composition. Studies addressing cachexia and frailty were ruled out.

In order to examine the studies’ methodological quality, the quality assessment tool adopted from the National Institutes of Health/National Heart, Lung and Blood Institute for observational cohort studies, cross-sectional studies, and case control studies was used (12). After answering the series of questions (14 regarding observational cohort studies and cross-sectional studies, 12 regarding case–control studies), the quality of cohort studies and cross-sectional studies was rated as poor (0–4 points), fair (5–10 points), or good (11–14 points); the quality of case–control studies was rated as poor (0–3 points), fair (4–8 points), or good (9–12 points).

## 3 Results

**Figure 1:**
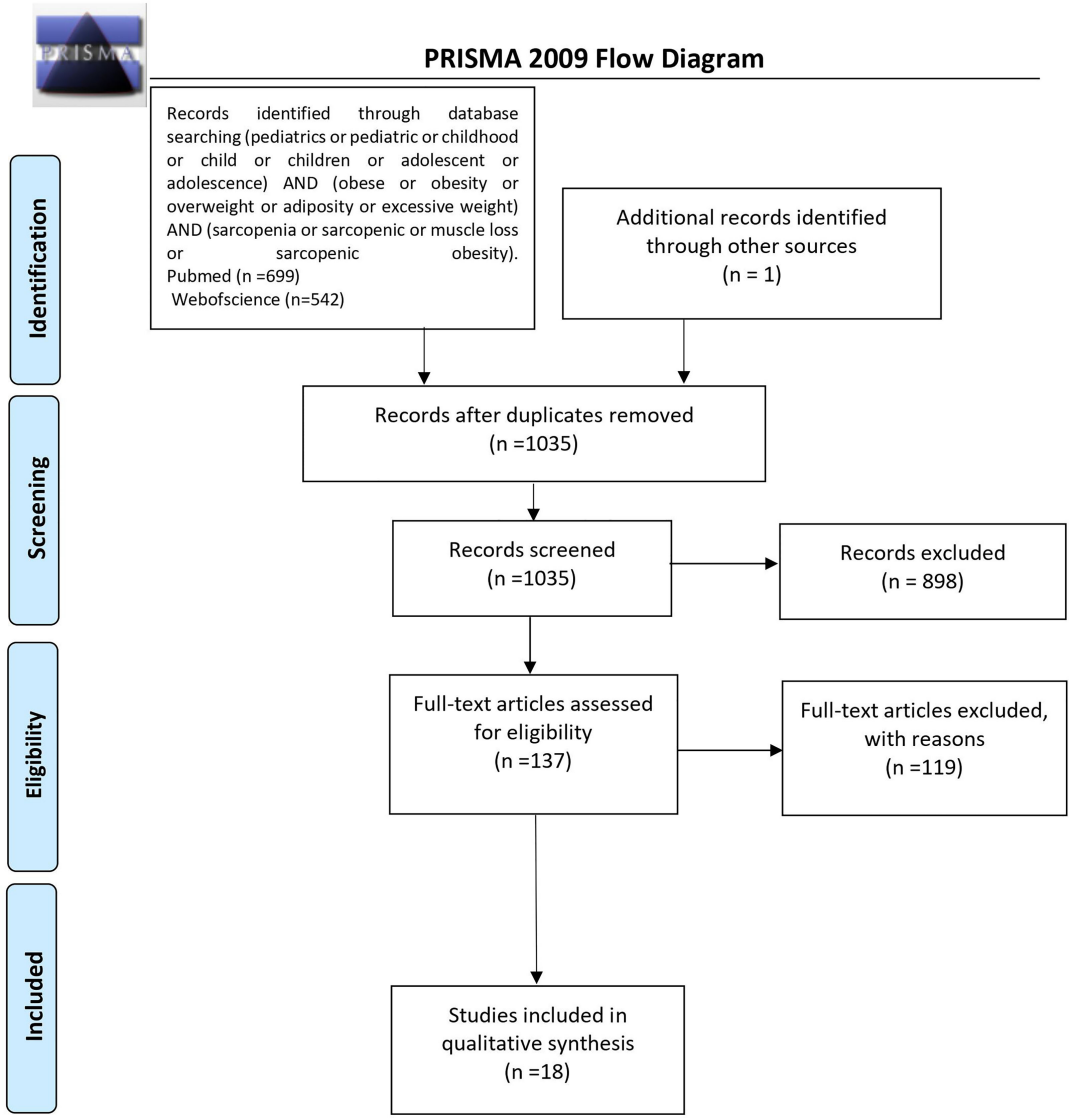
PRISMA protocol for data acquisition. n, number; PRISMA, Preferred Reporting Items for Systematic Reviews and Meta-Analyses (50).

The initial search returned 1,241 results. Additional studies were identified by a manual search of bibliographic references of existing reviews. The repeated results of search were ruled out. After screening of abstracts, 137 results were chosen for full-text analysis, of which 18 (25, 33–49) met the inclusion criteria and were included in the study. A flowchart of study inclusion is presented in **Figure 1**. The following information was abstracted from original papers: first author, geographic region, year of publication, time of the study, study design, number, sex, age of patients, study population, and data regarding SO definition, prevalence, and relation with outcomes. The characteristics of studies that included healthy populations are shown in **Table 1**, studies regarding overweight/obese children and adolescents are summarized in **Table 2**, and studies concerning other clinical populations are demonstrated in **Table 3**. Of 18 studies included in this review, 8 (25, 33–37, 42, 45) examined the prevalence of SO and 8 (33–35, 37, 43, 44, 47, 48) evaluated outcomes related to SO. Seven studies were performed in Europe (25, 34–36, 42, 45, 48), 5 in the United States (38, 40, 46, 47, 49), 3 in Asia (33, 37, 41), and 3 in South America (39, 43, 44). The number of participants ranged from 12 (46) to 15,392 (45). Based on the NIH quality assessment tool, all studies were classified as fair quality. Populations of studies by Kim et al. (33), Moon et al. (37), and Kim and Park (41) might be alike, since all of the studies used data from the Korean National Health and Nutrition Examination Survey (KHANES) conducted in similar years in subjects in the similar age range. However, all of the studies were included in this review, considering the fact that different definitions and cutoff values for SO were used in each of the studies, yielding different SO prevalence. The study by Orgel et al. (38) and the study by Mueske et al. (46) regarded patients newly diagnosed with high-risk B-acute lymphoblastic leukemia (ALL) or T-cell ALL who were prospectively enrolled in a clinical trial studying body composition and bone health. Nevertheless, both studies used different methods to evaluate sarcopenia, and therefore, both of the studies are part of this review. Both studies by Burrows et al. (43, 44) studied adolescents who were part of an iron deficiency anemia preventive trial and a follow-up study beginning in infancy. Although both studies used the same body composition evaluation method and sarcopenia and excessive weight indicators, SO definition differed between the aforementioned studies; therefore, neither of them were excluded from our review. Among the patients included in the 6 studies evaluating the prevalence of SO, the prevalence ranged from 5.66% (25) to 69.7% (45) in girls, with a range between 7.2% (42) and 81.3% (37) in boys.

**Table 1:** Characteristics of studies regarding healthy population.

Authors	Region	Year published	Time of study	Design	Number	Sex	Age, years	Population	Study quality (NIH)	Method of body composition evaluation	Sarcopenia indicator	Excessive weight indicator	Definition of SO	SO prevalence	Assessment of outcomes related to SO	Control group
Gontarev S et al. (36)	North Macedonia	2020	2017	CS	4021	49.4% male	range: 6-10 mean: 8.6	Healthy children from primary schools	6	BIA, dynamometer	MFR= SMM/BFM, grip-to-BMI ratio= maximal handgrip strength/BMI	NA	mean MFR-2SD of the 3rdBMI quintile/estimation of cut-off points of grip-to-BMI ratio	boys: 9.2% girls: 5.9% total: 7.5%	NA	no
Steffl M et al. (42)	Czech Republic	2017	2015	CS	730	51.64% male	range: 4-14	Healthy children and adolescents	6	BIA, dynamometer	MFR= SMM/BFM, grip-to-BMI ratio=maximal handgrip strength/BMI	NA	mean MFR-2SD of the 3rdBMI quintile)/estimation of cut-off points of grip-to-BMI ratio	boys: 7.2% girls: 9.3%	NA	no
Gätjens I et al. (45)	Germany	2021	since 1996	CS	15 392	49.38% male	range: 5-17	Healthy children and adolescents	6	BIA	FM/FFM, FM/FFM^2^	age and sex-specific reference percentiles of BMI in children and adolescents according to Kromeyer-Hauschild et al., 2001, BMI>90 th percentile of the study population	FM/FFM >90th percentile/FM/FFM^2^>90th percentile	boys: 62.7% girls: 69.7%	NA	no
McCarthy HD et al. (25)	UK	2013	2003-2004	CS	1985	56.22% male	range: 5-18.8	Healthy schoolchildren	6	BIA	MFR= SMMa/FM	highest fifth of BMI-z score for age range and sex	below mean MFR-2 SD of the middle fifth of the BMI range, highest fifth of BMI-z score for age range and sex	boys 5-10y: 8.31% boys 10-18y: 9.67% girls 5-10y: 15.48% girls 10-18y: 5.66%	NA	no
Stefanaki Ch et al. (48)	Italy	2016	2009-2012	CC	2551	lean group 16% females, overweight 95% females	range: 18-21	Healthy lean group, healthy overweight group	6	BIA	SMM	BMI between 25 and 35, fat mass as body weight percentage >25% for males and >32% for females	lower SMM in comparison with healthy lean group	NA	hsCRP, cortisol concentration at 8 a.m. and 8 p.m.	yes- gender and age range matched
Kim K et al. (33)	Republic of Korea	2016	2009-2011	CS	1919	53.36% male	range: 10-18	Healthy non-institutionalized Korean children and adolescents	7	DXA	MFR=ASM/body fat mass	BMI≥85th percentile for sex and age according to Standard Growth Charts of Korean children and adolescents published by the KCDC and Korean Pediatric Society in 2007, highest quintile of BMI	mean MFR-1SD of the 3rdBMI quintile	boys: 32.1% girls: 24.3%	Metabolic syndrome components (BP, glucose level, TG, HDL-C, WC)	no
Moon JH et al. (37)	Republic of Korea	2018	2008-2011	CS	1233	53.69% male	range:12-18	Healthy Korean adolescents	7	DXA	ASM, ASM/Wt(%)	WHtR>0.47 in both sexes	lower 10% of gender-specific ASM/Wt (%), WHtR>0.47 in both sexes	boys: 81.3% girls: 62.6%	Mental health	no
Kim JH et al. (41)	Republic of Korea	2016	2009-2011	CS	1420	52.75% male	range:12-19	Healthy Korean adolescents	7	DXA	ASM/Wt	WC at least 90th percentile for age and sex according to National Cholesterol Education Program-Adult Treatment Panel III Criteria	ASM/Wt below lower quintile for the study population, WC at least 90th percentile	NA	NA	no
Burrows R et al. (43)	Chile	2015	NA	CS	667	52.2% male	range:16-17 mean: 16.8	Healthy Chilean adolescents of middle to low SES	6	DXA	FFMI-estimated according to Wells and Fewtrell	BMI Z-score≥2 according to WHO, WC ≥80 cm in females, WC ≥ 90 cm in males	FFMI as percentage ≤25th percentile in sample (adjusted for sex), BMI Z-score≥2/WC ≥80 cm in females, WC ≥ 90 cm in males	NA	Metabolic syndrome components (BP, fasting serum total glucose, TG, HDL-C), insulin, HOMA-IR, cholesterol, adiponectin, hsCRP	no
Burrows R et al. (44)	Chile	2015	NA	CS	667	52.2% male	range:16-17 mean: 16.8	Healthy Chilean adolescents of middle to low SES	7	DXA	FFMI-estimated according to Wells and Fewtrell	BMI Z-score≥2 according to WHO,WC ≥80 cm in females, WC ≥ 90 cm in males	FFMI as percentage of BMI ≤25th percentile in sample (adjusted for sex),BMI Z-score≥2/WC ≥80 cm in females, WC ≥ 90 cm in males	NA	Metabolic syndrome components (BP, fasting serum total glucose, TG, HDL-C), insulin, HOMA-IR, cholesterol, adiponectin, hsCRP	no
**Palacio-Agüero A et al. (** 39)	Chile	2020	2018	CS	491	51.73% male	range: 10-17 mean: 13.6	Healthy Chilean adolescents	7	dynamometer	RHGS =maximum HGS from dominant hand/BMI	BMI-for-age value over +1 SD according to WHO, WC according to the guidelines from Chilean Ministry of Health	RHGS<25th percentile by sex, BMI-for-age over +1 SD/WC according to the guidelines from Chilean Ministry of Health	NA	NA	no

ASM, appendicular skeletal muscle mass; BFM, body fat mass; BIA, bioelectrical impedance analysis; BMI, body mass index; BP, blood pressure; CC, case–control; CS, cross-sectional; DXA, dual-energy X-ray absorptiometry; FFMI, fat-free mass index; FM, fat mass; FMI, fat mass index; HDL-c, high-density lipoprotein cholesterol; HGS, handgrip strength; HOMA-IR, homeostatic model assessment for insulin resistance; hsCRP, high-sensitivity C-reactive protein; MFR, skeletal muscle-to-body fat ratio; NA, not available; RHGS, relative handgrip strength; SES, socioeconomic status; SMM, skeletal muscle mass; SMMa, appendicular skeletal muscle mass; TG, triglyceride; WC, waist circumference; WHtR, waist circumference-to-height ratio; Wt, weight.

**Table 2:** Characteristics of studies concerning overweight/obese children and adolescents.

Authors	Region	Year published	Time of study	Design	Number	sex	Age, years	Population	Study quality (NIH)	Method of body composition evaluation	Sarcopenia indicator	Excessive weight indicator	Definition of SO	SO prevalence	Assessment of outcomes related to SO	Control group
Videira-Silva A et al. (34)	Portugal	2017	NA	R, CS	240	47.9% male	range: 10-17	Overweight adolescents attending a Pediatric Outpatient Obesity Clinic	8	BIA	%SMM=SMM/body weight MFR=SMM/BFM	BMI≥85th percentile for sex and age	%SMM≤p25 according to reference charts for youth McCarthy H.D. et al.	boys: 33.3% girls: 20,2% total: 26.9%	BP, glucose level, insulin level, HOMA-IR, total cholesterol, TG, HDL-C, LDL-C, CRP	no
Pacifico L et al. (35)	Italy	2020	NA	O, CS	234	56.41% male	range: 6-18	Overweight/obese children and adolescents attending Outpatient Clinics of the Department of Pediatrics	6	DXA	RMM=100x muscle mass/muscle mass+fat mass, ASM/weight index= ASM/weight x100	BMI> 85th percentile for sex and age	tertile 1 of RMM/tertile 1 of ASM/weight index	boys: 28.79% girls: 39.22% total: 33.33%	Metabolic syndrome components (WC, BP, glucose, HDL-C,TG),assessment of liver function(ALT, AST, liver US, liver biopsy),insulin, HOMA-IR, total cholesterol	no
Yodoshi T et al. (47)	USA	2020	2009-2018	R, CS	100 histology cohort, 263 liver stiffness cohort, 95 liver PDFF cohort	histology 65% male, liver stiffness 68% male, liver PDFF 77% male	<20	Patients with NAFLD	7	MRI	tPMSA index= tPMSA/height^2^	BMI≥85th percentile for sex and age, Centers for Disease Control and Prevention growth charts	lower median tPMSA index in comparison with subjects with NAS<5	NA	NAFLD activity score (NAS), liver stiffness, liver fat fraction	no

ALT, alanine aminotransferase; ASM, appendicular skeletal muscle mass; AST, aspartate aminotransferase; BFM, body fat mass; BIA, bioelectrical impedance analysis; BMI, body mass index; BP, blood pressure; CS, cross-sectional; CRP, C-reactive protein; DXA, dual-energy X-ray absorptiometry; HDL-c, high-density lipoprotein cholesterol; HOMA-IR, homeostatic model assessment for insulin resistance; LDL-c, low-density lipoprotein cholesterol; MFR, skeletal muscle-to-body fat ratio; MRI, magnetic resonance imagining; NA, not available; NAFLD, nonalcoholic fatty liver disease; NAS, NAFLD activity score; O, observational; PDFF, proton density fat fraction; R, retrospective; RMM, relative muscle mass; SMM, skeletal muscle mass; TG, triglyceride; tPMSA, total psoas muscle surface area; US, ultrasonography; WC, waist circumference.

**Table 3:** Characteristics of studies which included other clinical populations.

Authors	Region	Year published	Time of study	Design	Number	sex	Age, years	Population	Study quality (NIH)	Method of body composition evaluation	Sarcopenia indicator	Excessive weight indicator	Definition of SO	SO prevalence	Assessment of outcomes related to SO	Control group
Mangus RS et al. (40)	USA	2017	2002-2012	CC	81	35.8% male	≤18	Pediatric end-organ disease patients	7	CT	sarcopenic index= total psoas area/height^2^	visceral fat index, subcutaneous fat index	lower sarcopenic index compared to controls, higher visceral fat and subcutaneous fat index compared to controls	NA	NA	yes, 1:1 age- and gender -matched
Mueske NM et al. (46)	USA	2019	2011-2014	P	12	42% male	range: 10-21 mean: 14.4	Pre-adolescents, AYA diagnosed with HR B-ALL or T-cell ALL	9	QCT, DXA	muscle volume/percent in the lower leg	BMI%≥85th according to Centers for Disease Control and Prevention before treatment initiation, increase of total body fat mass/fat percent during therapy	loss of muscle volume/muscle percent over time, increase of total body fat mass/fat percent over time	NA	NA	yes- age range matched
Joffe L et al. (49)	USA	2020	2002-2017	R	39	53.8% female	range: 1.33-20 mean: 9.8 median:11	Children, adolescents and young adults with solid tumors	10	CT	SM,RLT	BMI≥85th percentile for sex and age WHO and CDC increase of VAT during therapy	loss of SM and RLT over time, increase of VAT over time	NA	NA	no
Orgel E et al. (38)	USA	2018	NA	P	50	60% male	range: 9.9-19.6 mean: 14.7 median:14.6	Children and adolescents diagnosed with High-Risk B-Precursor ALL or T-cell ALL	7	DXA	Lean muscle mass	BMI≥85th percentile for sex and age according to CDC criteria before treatment initiation, increase of BF% during therapy	loss of lean muscle mass over time, increase of BF% over time	NA	NA	no

ALL, acute lymphoblastic leukemia; AYA, adolescents and young adults; BF, body fat; BMI, body mass index; CC, case–control; CDC, Centers for Disease Control and Prevention; CT, computed tomography; DXA, dual-energy X-ray absorptiometry; NA, not available; P, prospective; QCT, quantitative computed tomography; R, retrospective; RLT, residual lean tissue; SM, skeletal muscle; VAT, visceral adipose tissue; WHO, World Health Organization.

### 3.1 Sarcopenic Obesity Evaluation Methods

*Overall* DXA (n = 8/18) (33, 35, 37, 38, 41, 43, 44, 46) and BIA (n = 6/18) (25, 34, 36, 42, 45, 48) were the most commonly used body composition evaluation methods, followed by other *imaging* techniques (4/18) (40, 46, 47, 49) and assessment of handgrip strength (HGS) using dynamometer (39). Three studies used more than one method to assess SO [BIA along with dynamometer (36, 42) and quantitive computed tomography (QCT) in addition to DXA (46)]. Overall, there was a wide heterogenity in the cutoff values used to define sarcopenia.

#### 3.1.1 Healthy Populations

In total, 11 studies included healthy populations of children and adolescents (25, 33, 36, 37, 39, 41–45, 48). Five studies used BIA to evaluate body composition (25, 36, 42, 45, 48), 5 studies used DXA (33, 37, 41, 43, 44), whereas 1 study was conducted with the use of the handgrip test (39).

##### 3.1.1.1 Bioelectrical Impedance

In the study by McCarthy et al. (25), muscle-to-fat ratio (MFR) was derived by dividing appendicular skeletal muscle mass (SMMa) by fat mass (FM). For each age range, in boys and in girls separately, children were divided into fifths of BMI z-score, and the mean and standard deviation (SD) of MFR within each fifth were calculated. MFR cutoff equating to -2 SD for the middle fifth was introduced for the first time, and SO was considered as a proportion of cases falling below the cutoff in the highest fifth of BMI z-score.

In the study by Stefanaki et al. (48), patients were divided into two groups: healthy lean group and healthy overweight/obese group. Lower SMM in comparison to healthy lean group for each gender separately was used as sarcopenia indicator.

In the study by Gätjens et al. (45), fat mass (FM) and fat-free mass (FFM) were normalized to height^2^ to fat mass index (FMI) and fat-free mass index (FFMI). Sarcopenic obese phenotype was defined as >90th percentile of FM/FFM or FM/FFM^2^.

Studies by Gontarev et al. (36) and Steffl et al. (42) assessed muscle strength using a dynamometer along with bioelectrical impedance from which MFR was calculated. The purpose of those studies was to determine the relationships between MFR and relative handgrip strength (RHGS) and to determine the ability of HGS relative to BMI (grip-to-BMI) to identify children who are at risk of developing SO. In both studies, a previous methodology used to define sarcopenia in children described by McCarthy et al. (25) and Kim et al. (33) was used. Results indicated that grip-to-BMI ratio is capable of identifying children at risk of SO.

##### 3.1.1.2 Dual-Energy X-Ray Absorptiometry

Studies by Moon et al. (37) and Kim and Park (41) used ASM divided by weight (ASM/Wt%) as a sarcopenia indicator. In the study by Moon et al. (37), LMM was defined as the lower 10% of gender-specific ASM/Wt%. Whereas in the study by Kim et al. (41), LMM was observed if the value for ASM/Wt was below the lower quintile for the study population; LMM cutoff points were calculated for each sex and age.

Two studies by Burrows et al. (43, 44) used FFMI estimated according to Wells and Fewtrell as a sarcopenia indicator. In the study by Burrows et al. (43), FFMI values were expressed as percentage; values ≤25th percentile in the study sample, adjusted for sex, were defined as relative sarcopenia. While in the study by Burrows et al. (44), FFMI values were expressed as the percentage of BMI; values ≤25th percentile in study sample, adjusted for sex, were considered sarcopenia.

In the study by Kim et al. (33), MFR was calculated, each gender was divided into quintiles of BMI z-score, and the mean and SD of MFR were calculated for each quintile; cutoff values were defined using the mean and SD of MFR for the third BMI quintile (cutoff value = mean value -1 SD of MFR for the third BMI quintile).

##### 3.1.1.3 Handgrip Test

In the study by Palacio-Agüero et al. (39), RHGS was calculated by dividing maximum HGS from the dominant hand by BMI. Low RHGS was defined as <25th percentile by sex.

#### 3.1.2 Overweight/Obese Populations

Overall, 3 studies involved overweight/obese children and adolescents (34, 35, 47).

In the study by Videira-Silva and Fonseca (34), %SMM ≤25 percentile based on reference charts for youth by McCarthy et al. (25) was used as a cutoff value for sarcopenia; BIA was used to assess the concept of SO.

In the study by Pacifico et al. (35), DXA was used to assess body composition, population was stratified into tertiles of relative muscle mass (RMM) and ASM/weight index, and children in the lowest tertiles were considered sarcopenic.

In the study by Yodoshi et al. (47), abdominal MRI at the level of the second to third lumbar vertebrae allowing the determination of total psoas muscle surface area (tPMSA) of patients with either histologically confirmed NAFLD with negative workup for other liver disease, and an abdominal MRI within 1 year of the liver biopsy, or presumed NAFLD, defined as overweight/obese patients with MRI-determined hepatic steatosis and a negative workup for other liver diseases, was evaluated. Patients with presumed NAFLD were divided into two groups: one consisted of patients who underwent magnetic resonance (MR) elastography to assess liver stiffness, and patients in group 2 had measured liver fat fraction by MRI proton density fat fraction (PDFF). tPMSA was measured using a geometric region of interest measurement tool (Intellispace). tPMSA was corrected for height, generating tPMSA index (mm^2^/m^2^). Patients with a lower median tPMSA index compared to patients with a lower grade (<5) of NAFLD activity score (NAS) according to non-alcoholic steatohepatitis (NASH) Clinical Research Network were identified as sarcopenic.

#### 3.1.3 Other Populations

The populations of studies by Orgel et al. (38) and Mueske et al. (46) consisted of children with hematologic malignancies (high-risk B-precursor ALL and T-cell ALL).

In the study by Orgel et al. (38), the concept of SO was evaluated with DXA; subjects underwent three serial assessments of body composition (at diagnosis, at the end of the induction phase, and at the end of the delayed intensification phase), and lean muscle mass was measured. SO was defined as loss of lean muscle mass and increase of body fat mass and body fat percent over time.

In the study by Mueske et al. (46), participants underwent imaging using three-dimensional QCT of the tibia and whole-body DXA within 96 h from the start of chemotherapy, again at 28–35 days later (end of induction phase), and 7–9 months from diagnosis following completion of intensive chemotherapy (end of the delayed intensification phase). Tissue volumes for adipose, muscle, and bone were computed along the entire length of both tibias using a custom MATLAB script. Total body fat mass and percentage were obtained using DXA. Loss of muscle volume/muscle percent and increase of total body fat mass/fat percent over time was considered as an SO indicator.

In the study by Joffe et al. (49), chest CT images obtained at two time points, diagnosis and first follow-up disease evaluation (6–14 weeks after initiation of therapy), of children, adolescents, and young adults who underwent treatment for a primary diagnosis of Wilms tumor, Ewing sarcoma, osteosarcoma, or rhabdomyosarcoma were used. Measurement of SM, residual lean mass (RLT), and visceral adipose tissue (VAT) was performed on single-slice images at a select anatomic landmark located in the intervertebral space between the 12th thoracic and first lumbar vertebrae (T12–L1). Image analysis was performed utilizing Slice-O-Matic image analysis software. SO was defined as a decrease in SM and RLT and increase of VAT between two study points.

In the study by Mangus et al. (40), body composition of children on the kidney, liver, or intestine transplant list with end-organ failure and a CT scan within 6 months of actual transplant date were assessed with measurements taken at the level of the L2/L3 intervertebral disc space. The scan was set to the soft tissue image mode, and the FreehandDrawingTool was employed to outline the target structures using Synapse picture archiving and communication system (PACS). Total psoas muscle area was obtained by outlining both the right and left psoas muscles and summing these measurements. Total perinephric fat was calculated by outlining the kidney and vasculature and subtracting this area from the area obtained by outlining Gerota’s fascia. The subcutaneous fat area was obtained by subtracting the area of the outlined abdominal cavity (at the outermost fascial layer) from the area obtained by outlining the level just beneath the dermis. The sarcopenic index was obtained by dividing the total psoas area (in mm^2^) by the height (in cm) squared, and the visceral and subcutaneous fat measurements were also scaled for height. SO was defined as lower sarcopenic index and higher visceral fat and subcutaneous fat index compared to controls.

### 3.2 Sarcopenic Obesity Prevalence

Eight studies included in the review assessed the prevalence of SO (25, 33–37, 42, 45); 6 studies included healthy populations (25, 33, 36, 37, 42, 45), whereas 2 studies examined overweight/obese children and adolescents (34, 35).

#### 3.2.1 Healthy Populations

Among the patients included in the studies evaluating healthy populations, the prevalence ranged from 5.66% (25) to 69.7% (45) in girls, with a range between 7.2% (42) and 81.3% (37) in boys. Evaluation of body composition using BIA with usage of mean MFR-2SD of the third BMI quintile as threshold, which was used in 3 studies (25, 36, 42), was associated with the lowest SO prevalence in boys [7.2% (42), 8.31% in boys 5–10 years (25), 9.2% (36), 9.67% in boys 10–18 years (25)] and in girls [5.66% in girls 10–18 years (25), 5.9% (36), 9.3% (42), 15.48% in girls 5–10 years (25)]. This was followed by a study that used DXA and a cutoff value defined as mean value -1 SD of MFR for the third BMI quintile, which yielded 24.3% SO prevalence in girls and 32.1% in boys (33).

Highest prevalence of SO was found in the usage of DXA and the lower 10% of gender-specific ASM/Wt% in boys (81.3%) (37) and using BIA along with >90th percentile of FM/FFM or FM/FFM^2^ in girls (69.7%) (45).

#### 3.2.2 Overweight/Obese Populations

Two studies evaluated SO prevalence in overweight/obese children and adolescents, the study that used DXA to assess RMM and ASM and stratified study population into tertiles of RMM and ASM/weight index yielded 28.79% SO prevalence in boys and 39.22% in girls (35), whereas the study by Videira-Silva and Fonseca (34) that used BIA and a cutoff value defined as %SMM ≤25 percentile based on reference charts for youth by McCarthy et al. (25) yielded 20.2% prevalence of SO in girls and 33.3% in boys.

### 3.3 Outcomes Related With Sarcopenic Obesity

A total of 8 (33–35, 37, 43, 44, 47, 48) studies assessed outcomes related to SO. Of those, 5 studies evaluated SO relation with cardiometabolic outcomes (33–35, 43, 44), and 2 studies assessed the association between NAFLD severity and presence of SO (35, 47). Two studies evaluated the relation between SO and inflammation (34, 48). The remaining study investigated the relationship between LMM, obesity, and mental health (37).

In 2 of the studies analyzing the occurrence of metabolic syndrome (MetS) (33, 43), MetS diagnosis was based on 2007 International Diabetes Federation (IDF) consensus, whereas in one study (35), MetS diagnosis was based on the presence of at least 3 risk factors: high waist circumference (WC), elevated blood pressure (BP), low high-density lipoprotein cholesterol (HDL-C) levels, hypertriglyceridemia, and glucose impairment. In all studies evaluating associations between sarcopenia, obesity, and MetS, significant associations were observed. Odds ratios (ORs) of MetS risk were found to be significantly increased in sarcopenic obese individuals [OR 8.28, 95% CI 5.6–11.45 (33); sarcopenic boys, OR 21.2, 95% CI 4.18–107.5 (43); obese boys, OR 3.7, 95% CI 1.23–10.8 (43); and sarcopenic girls, OR 3.61, 95% CI 1.10–11.9) (43)]; the prevalence of MetS was also significantly increased in the tertile 1 of RMM (p < 0.0001) (35).

Remaining studies that analyzed SO association with cardiometabolic outcomes showed significant associations in terms of insulin resistance (IR) and obesity (OR 6.6, 95% CI 4.1–10.6) (44) and IR and sarcopenia (OR 4.9, 95% CI 3.2–7.5) in bivariate analysis (44), IR and sarcopenia (OR 1.9, 95% CI 1.1–3.6) and IR and obesity (OR 2.4, 95% CI 1.2–4.9) in the fully adjusted model (including family history of type 2 diabetes, physical inactivity, sarcopenia, obesity, low adiponectin) (44), increased insulin (p < 0.01) (34), homeostatic model assessment for insulin resistance (HOMA-IR) (p < 0.05) (34, 35), total cholesterol (p < 0.05) (34), low-density lipoprotein cholesterol (LDL-C) (p < 0.01) (34), triglyceride (TG) (p < 0.05) (34, 35), decreased HDL-C (p < 0.05) (34, 35), and NAFLD (p < 0.05) (35).

In 2 studies (35, 47) aiming to assess NAFLD severity in terms of SO, children with a higher severity of NAFLD [NAS/liver PDFF/non-alcoholic steatohepatitis (NASH) occurrence] had lower values of sarcopenia indicator (tPMSA index/RMM/ASM/weight) in comparison with the ones with lower liver disease severity. In the study by Pacifico et al. (35), children with NASH showed significantly lower RMM [mean 55.7% (SD, 6.0) *vs*. 63.4% (6.0); p < 0.0001) and ASM/weight index [mean 25.6% (SD, 2.8) *vs*. 28.6% (2.9); p = 0.006], whereas in the histology cohort from the study of Yodoshi et al. (47), median tPMSA index was significantly lower in the subjects with NAS ≥5 compared to those with NAS <5 (544 mm^2^/m^2^ *vs*. 669 mm^2^/m^2^, p < 0.001). In both univariate logistic regression analysis with proportional odds and multivariable analysis (including all demographic, clinical, radiographic variables), higher NAS was significantly associated with lower tPMSA index (OR 0.67, 95% CI 0.52–0.86, p = 0.002) and tPMSA index significantly predicted NAS (OR 0.68, 95% CI 0.52–0.91, p = 0.008), respectively. Moreover, in the liver PDFF cohort in the multivariable regression model (including tPMSA index, sex, ethnicity, community deprivation index, and T2DM), liver PDFF was significantly associated with tPMSA index (p = 0.029) (47).

Both studies (34, 48) aiming to investigate SO relation with inflammation, an assessment of C-reactive protein (CRP)/high-sensitivity C-reactive protein (hsCRP) concentration, showed significant associations. In the study by Videira-Silva and Fonseca (34), a low value of SMM (%SMM ≤p25) was associated with an increased value of inflammation indicator (p < 0.05), whereas in the study by Stefanaki et al. (48), SMM was negatively correlated with hsCRP concentration in overweight/obese individuals.

In the study by Moon et al. (37), girls with LMM and obesity were 3.46 times more at risk of developing depression compared with girls with normal muscle mass after controlling for age, waist-to-height ratio, health habits, self-reported obesity, weight loss efforts, and monthly household income (95% CI 1.00–11.97, p = 0.049).

## 4 Discussion

There are a limited number of studies assessing the prevalence of SO and its relation with adverse health outcomes in children and adolescents. Results of our review indicate that SO is highly prevalent in children and adolescents and influences the occurrence of adverse health outcomes (25, 33–37, 42–45, 47, 48).

In our review, the prevalence of SO ranged from 5.66% to 69.7% in girls, with a range between 7.2% and 81.3% in boys. The wide range of SO prevalence is related to different sarcopenia evaluation methods and the variety of used definitions and cutoff points. Furthermore, studies that evaluated SO prevalence included healthy populations and overweight/obese children and adolescents; therefore, comparisons between those populations may lead to discrepancies, as studies regarding overweight/obese participants may yield higher SO prevalence.

Our review found DXA and BIA to be the most prevalent methods of body composition assessment. Evaluation of body composition with usage of mean MFR-2SD of the third BMI quintile as threshold was the most prevalent. All of the studies that assessed SO relation with adverse health outcomes (cardiometabolic outcomes, NAFLD severity, inflammation, mental health) found significant associations. The main difficulty regarding the assessment of sarcopenia in the population of children and adolescents is their growth, which leads to age-related differences. Puberty is also a crucial factor in body composition evaluation. The present review indicates that the occurrence of SO is associated with a higher metabolic risk. Much work needs to be done to understand the impact of SO on metabolic outcomes, as SO may carry a cumulative metabolic risk of both sarcopenia and obesity and could lead to worse metabolic outcomes than obesity alone. Routine assessment of SM mass and function should be considered in obese children and adolescents in order to distinguish individuals with a higher metabolic risk.

Only 2 studies included in our review assessed both muscle mass and muscle function (36, 42), as recommended by EWGSOP, yielding inadequate values of SO prevalence. The assessment of muscle function is crucial for the assessment of sarcopenia, since muscle strength is not linearly related to muscle mass (30).

SO has been associated with various adverse health outcomes such as disability, metabolic outcomes, depression, increased stress level, cancer treatment outcomes, and mortality in adults (16). Several studies evaluated SO association with metabolic outcomes in adults, indicating worse cardiovascular risk profiles (hyperglycemia, hypertension, dyslipidemia, insulin resistance, lower cardiorespiratory fitness) in individuals affected by SO (51–53). Results of the Korean Longitudinal Study on Health and Aging (KLoSHA) demonstrated a higher risk of IR and MetS in patients with SO than in those with sarcopenia alone or obesity alone (51). According to KHANES, SO was also associated with NAFLD, and the study found that SO increased stepwise from lowest to highest quintile (independent 3.4-fold risk) of serum gamma-glutamyl transferase activity (GGT) in community-dwelling older adults (54). NASH as a cause of cirrhosis was found in multivariable logistic regression analysis as an independent predictor of SO after controlling for age, gender, alcoholic liver disease diagnosis, and hepatocellular carcinoma (p = 0.014, 95% CI, 1.44–25.26, OR 6.03) (55). In a study by Schrager et al. (56), low HGS and high waist circumference and/or BMI were significantly associated with elevated levels of IL-6, C-reactive protein, and Interleukin-1 (IL-1). In a study by Hamer et al. (57), the risk of occurrence of depressive symptoms in obese adults with low HGS was 1.79 (95% CI, 1.10–2.89) times greater compared to non-obese individuals with high HGS after multivariate adjustment. These findings of adult studies are in line with results of our review, which demonstrated a significant association between adverse health outcomes (metabolic syndrome, IR, NAFLD) and SO in children and adolescents. Moreover, in two studies included in our review, low SMM demonstrated an association with increased hsCRP/CRP concentration, indicating a relation between inflammation and low SMM in overweight/obese children and adolescents. Results of our review regarding the association between LMM, obesity, and depressive symptoms also match adult studies.

To our knowledge, this is the first systematic review evaluating the prevalence and outcomes of SO in children and adolescents. Our study is not without limitations. There was considerable inconsistency in the criteria used to define SO, and there was a wide heterogeneity in sarcopenia as well as obesity definitions that limited comparisons among studies. Furthermore, the number of study participants varied between the studies that also led to difficulties regarding comparisons. Included studies differed in terms of included populations (healthy populations, overweight/obese participants, other clinical populations) that yielded further difficulties regarding data comparability.

Consensus regarding SO definition and implementation of standardized evaluation methods in children and adolescents should be reached in order to conduct studies assessing the exact prevalence of SO and its impact on outcomes. Furthermore, according to EWSOGP2, low muscle strength is a primary sarcopenia parameter and should always be evaluated next to muscle mass. Further studies providing age and gender thresholds for SO for different ethnicities are needed.

## 5 Conclusions

In conclusion, in our review, the prevalence of SO ranged from 5.66% to 69.7% in girls, with a range between 7.2% and 81.3% in boys. Association between SO and various adverse health outcomes was found. Considering the fact that no consensus regarding SO definition in children and adolescents has been reached and studies included in our review used a wide range of methods and definitions to evaluate the presence of SO, results of our review concerning SO prevalence and adverse health outcomes related to SO might not be adequate. Findings of this review highlight the need for the development of a consensus regarding definition, standardized evaluation methods, and age and gender thresholds for SO for different ethnicities in the pediatric population in order to produce meaningful results and implications for clinical practice. Routine assessment of SM mass and function in obese pediatric patients should be taken into consideration. Moreover, effective treatment strategies for children and adolescents with SO should be developed, as SO might play a role in the occurrence of adverse health outcomes.

## Data Availability Statement

The original contributions presented in the study are included in the article/supplementary material. Further inquiries can be directed to the corresponding author.

## Author Contributions

Conceptualization: MZ and PM. Methodology: MZ and PM. Validation: MZ and PM. Data analysis: MZ. Writing—original draft preparation: MZ. Writing—review and editing: PM. Supervision: PM. Project administration: MZ and PM. All authors have read and agreed to the published version of the article.

## Conflict of Interest

The authors declare that the research was conducted in the absence of any commercial or financial relationships that could be construed as a potential conflict of interest.

## Publisher’s Note

All claims expressed in this article are solely those of the authors and do not necessarily represent those of their affiliated organizations, or those of the publisher, the editors and the reviewers. Any product that may be evaluated in this article, or claim that may be made by its manufacturer, is not guaranteed or endorsed by the publisher.

## References

[1] WHO . Overweight and Obesity. Available at: https://www.who.int/news-room/fact-sheets/detail/obesity-and-overweight (Accessed 1.12.2021).

[2] HEALTHY Study Group Kaufman FR Hirst K Linder B Baranowski T Cooper DM . Risk Factors for Type 2 Diabetes in a Sixth- Grade Multiracial Cohort: The sHEALTHY Study. Diabetes Care (2009) 32(5):953–5. doi: 10.2337/dc08-1774 PMC2671115 19196888

[3] Sorof J Daniels S . Obesity Hypertension in Children: A Problem of Epidemic Proportions. Hypertension (2002) 40:441–7. doi: 10.1161/01.HYP.0000032940.33466.12 12364344

[4] Kumar S Kelly AS . Review of Childhood Obesity: From Epidemiology, Etiology, and Comorbidities to Clinical Assessment and Treatment. Mayo Clin Proc (2017) 92(2):251–65. doi: 10.1016/j.mayocp.2016.09.017 28065514

[5] Feldstein AE Charatcharoenwitthaya P Treeprasertsuk S Benson JT Enders FB Angulo P . The Natural History of non-Alcoholic Fatty Liver Disease in Children: A Follow-Up Study for Up to 20 Years. Gut (2009) 58:1538–44. doi: 10.1136/gut.2008.171280 PMC2792743 19625277

[6] Spilsbury JC Storfer-Isser A Rosen CL Redline S . Remission and Incidence of Obstructive Sleep Apnea From Middle Childhood to Late Adolescence. Sleep (2015) 38:23–9. doi: 10.5665/sleep.4318 PMC4262952 25325456

[7] Strauss RS . Childhood Obesity and Self-Esteem. Pediatrics (2000) 105:e15. doi: 10.1542/peds.105.1.e15 10617752

[8] Rosenberg I . Summary Comments: Epidemiological and Methodological Problems in Determining Nutritional Status of Older Persons. Am J Clin Nutr (1989) 50:1231–3. doi: 10.1093/ajcn/50.5.1231

[9] Rosenberg IH . Sarcopenia: Origins and Clinical Relevance. J Nutr (1997) 127:990S–91S. doi: 10.1093/jn/127.5.990S 9164280

[10] Cruz-Jentoft AJ Baeyens JP Bauer JM Boirie Y Cederholm T Landi F . Sarcopenia: European Consensus on Definition and Diagnosis: Report of the European Working Group on Sarcopenia in Older People. Age Ageing (2010) 39(4):412–23. doi: 10.1093/ageing/afq034 PMC2886201 20392703

[11] Cruz-Jentoft AJ Bahat G Bauer J Boirie Y Bruyère O Cederholm T . Sarcopenia: Revised European Consensus on Definition and Diagnosis [Published Correction Appears in Age Ageing. Age Ageing (2019) 48(1):16–31. doi: 10.1093/ageing/afy169 30312372 PMC6322506

[12] Woo J. Sarcopenia. Clin Geriatr Med (2017) 33(3):305–14. doi: 10.1016/j.cger.2017.02.003 28689564

[13] Koliaki C Liatis S Dalamaga M Kokkinos A . Sarcopenic Obesity: Epidemiologic Evidence, Pathophysiology, and Therapeutic Perspectives. Curr Obes Rep (2019) 8(4):458–71. doi: 10.1007/s13679-019-00359-9 31654335

[14] Sayer AA Syddall H Martin H Patel H Baylis D Cooper C . The Developmental Origins of Sarcopenia. J Nutr Health Aging (2008) 12(7):427–32. doi: 10.1007/BF02982703 PMC2652119 18615224

[15] Gallagher D Belmonte D Deurenberg P Wang Z Krasnow N Pi-Sunyer FX . Organ-Tissue Mass Measurement Allows Modeling of REE and Metabolically Active Tissue Mass. Am J Physiol (1998) 275(2):E249–58. doi: 10.1152/ajpendo.1998.275.2.E249 9688626

[16] Batsis JA Villareal DT . Sarcopenic Obesity in Older Adults: Aetiology, Epidemiology and Treatment Strategies. Nat Rev Endocrinol (2018) 14(9):513–37. doi: 10.1038/s41574-018-0062-9 PMC6241236 30065268

[17] Benson AC Torode ME Singh MA . Muscular Strength and Cardiorespiratory Fitness is Associated With Higher Insulin Sensitivity in Children and Adolescents. Int J Pediatr Obes (2006) 1:222–31. doi: 10.1080/17477160600962864 17907329

[18] Steene-Johannessen J Anderssen SA Kolle E Andersen LB . Low Muscle Fitness is Associated With Metabolic Risk in Youth. Med Sci Sports Exerc (2009) 41:1361–7. doi: 10.1249/MSS.0b013e31819aaae5 19516166

[19] Chien MY Huang TY Wu YT . Prevalence of Sarcopenia Estimated Using a Bioelectrical Impedance Analysis Prediction Equation in Community-Dwelling Elderly People in Taiwan. J Am Geriatr Soc (2008) 56:1710–5. doi: 10.1111/j.1532-5415.2008.01854.x 18691288

[20] Gilligan LA Towbin AJ Dillman JR Somasundaram E Trout AT . Quantification of Skeletal Muscle Mass: Sarcopenia as a Marker of Overall Health in Children and Adults. Pediatr Radiol (2020) 50:455–64. doi: 10.1007/s00247-019-04562-7 31745597

[21] Buckinx F Landi F Cesari M Fielding RA Visser M Engelke K . Pitfalls in the Measurement of Muscle Mass: A Need for a Reference Standard. J Cachexia Sarcopenia Muscle (2018) 9:269–78. doi: 10.1002/jcsm.12268 PMC5879987 29349935

[22] Janssen I Heymsfield SB Baumgartner RN Ross R . Estimation of Skeletal Muscle Mass by Bioelectrical Impedance Analysis. J Appl Physiol (2000) 89:465–71. doi: 10.1152/jappl.2000.89.2.465 10926627

[23] Ooi PH Hager A Mazurak VC Dajani K Bhargava R Gilmour SM . Sarcopenia in Chronic Liver Disease: Impact on Outcomes. Liver Transpl (2019) 25(9):1422–1438. doi: 10.1002/lt.25591 31242345

[24] Kim KM Jang HC Lim S . Differences Among Skeletal Muscle Mass Indices Derived From Height-, Weight-, and Body Mass Index-Adjusted Models in Assessing Sarcopenia. Korean J Intern Med (2016) 31:643–50. doi: 10.3904/kjim.2016.015 PMC4939509 27334763

[25] McCarthy HD Samani-Radia D Jebb SA Prentice AM . Skeletal Muscle Mass Reference Curves for Children and Adolescents. Pediatr Obes (2014) 9(4):249–59. doi: 10.1111/j.2047-6310.2013.00168.x 23776133

[26] Guo B Wu Q Gong J Xiao Z Tang Y Shang J . Relationships Between the Lean Mass Index and Bone Mass and Reference Values of Muscular Status in Healthy Chinese Children and Adolescents. J Bone Miner Metab (2016) 34:703–13. doi: 10.1007/s00774-015-0725-8 26586459

[27] Schmidt SC Bosy-Westphal A Niessner C Woll A . Representative Body Composition Percentiles From Bioelectrical Impedance Analyses Among Children and Adolescents. MoMo Study Clin Nutr (2019) 38(6):2712–20. doi: 10.1016/j.clnu.2018.11.026 30554799

[28] Webber CE Barr RD . Age- and Gender-Dependent Values of Skeletal Muscle Mass in Healthy Children and Adolescents. J Cachexia Sarcopenia Muscle (2012) 3:25–9. doi: 10.1007/s13539-011-0042-6 PMC3302981 22451073

[29] Been E Shefi S Kalichman L Bailey JF Soudack M . Cross-Sectional Area of Lumbar Spinal Muscles and Vertebral Endplates: A Secondary Analysis of 91 Computed Tomography Images of Children Aged 2-20. J Anat (2018) 233(3):358–69. doi: 10.1111/joa.12838 PMC6081509 29926903

[30] Merli M . Pediatric Sarcopenia: Exploring a New Concept in Children With Chronic Liver Disease. J Pediatr (2020) 96(4):406–8. doi: 10.1016/j.jped.2019.08.001 PMC9432108 31469973

[31] Griffiths A Toovey R Morgan PE Spittle AJ . Psychometric Properties of Gross Motor Assessment Tools for Children: A Systematic Review. BMJ Open (2018) 8(10):e021734. doi: 10.1136/bmjopen-2018-021734 PMC6224743 30368446

[32] Hardin AP Hackell JM . COMMITTEE ON PRACTICE AND AMBULATORY MEDICINE. Age Limit Pediatr Pediatr (2017) 140(3):e20172151. doi: 10.1542/peds.2017-2151 28827380

[33] Kim K Hong S Kim EY . Reference Values of Skeletal Muscle Mass for Korean Children and Adolescents Using Data From the Korean National Health and Nutrition Examination Survey 2009-2011. PloS One (2016) 11(4):e0153383. doi: 10.1371/journal.pone.0153383 27073844 PMC4830599

[34] Videira-Silva A Fonseca H . Skeletal Muscle and Metabolic Risk in Overweight Adolescents. An Indicator of Premature Sarcopenic Obesity. Int J Health Sci (2017) 7:34–43.

[35] Pacifico L Perla FM Andreoli G Grieco R Pierimarchi P Chiesa C . Nonalcoholic Fatty Liver Disease Is Associated With Low Skeletal Muscle Mass in Overweight/Obese Youths. Front Pediatr (2020) 8:158. doi: 10.3389/fped.2020.00158 32351917 PMC7174581

[36] Gontarev S Jakimovski M Georgiev G . Using Relative Handgrip Strength to Identify Children at Risk of Sarcopenic Obesity. Nutr Hosp (2020) 34(3):490–6. doi: 10.20960/nh.02977 32406745

[37] Moon JH Kong MH Kim HJ . Low Muscle Mass and Depressed Mood in Korean Adolescents: A Cross-Sectional Analysis of the Fourth and Fifth Korea National Health and Nutrition Examination Surveys. J Korean Med Sci (2018) 33(50):e320. doi: 10.3346/jkms.2018.33.e320 30534032 PMC6281954

[38] Orgel E Mueske NM Sposto R Gilsanz V Freyer DR Mittelman SD . Limitations of Body Mass Index to Assess Body Composition Due to Sarcopenic Obesity During Leukemia Therapy. Leuk Lymphoma (2018) 59(1):138–45. doi: 10.3109/10428194.2015.1136741 PMC5362342 26818609

[39] Palacio-Agüero A Díaz-Torrente X Quintiliano Scarpelli Dourado D . Relative Handgrip Strength, Nutritional Status and Abdominal Obesity in Chilean Adolescents. PloS One (2020) 15(6):e0234316. doi: 10.1371/journal.pone.0234316 32520942 PMC7286492

[40] Mangus RS Bush WJ Miller C Kubal CA . Severe Sarcopenia and Increased Fat Stores in Pediatric Patients With Liver, Kidney, or Intestine Failure. J Pediatr Gastroenterol Nutr (2017) 65(5):579–83. doi: 10.1097/MPG.0000000000001651 28604513

[41] Kim JH Park YS . Low Muscle Mass is Associated With Metabolic Syndrome in Korean Adolescents: The Korea National Health and Nutrition Examination Survey 2009-2011. Nutr Res (2016) 36(12):1423–8. doi: 10.1016/j.nutres.2016.09.013 27884414

[42] Steffl M Chrudimsky J Tufano JJ . Using Relative Handgrip Strength to Identify Children at Risk of Sarcopenic Obesity. PloS One (2017) 12(5):e0177006. doi: 10.1371/journal.pone.0177006 28542196 PMC5441624

[43] Burrows R Correa-Burrows P Reyes M Blanco E Albala C Gahagan S . High Cardiometabolic Risk in Healthy Chilean Adolescents: Associations With Anthropometric, Biological and Lifestyle Factors. Public Health Nutr (2016) 19(3):486–93. doi: 10.1017/S1368980015001585 PMC4654715 25990645

[44] Burrows R Correa-Burrows P Reyes M Blanco E Albala C Gahagan S . Healthy Chilean Adolescents With HOMA-IR ≥ 2.6 Have Increased Cardiometabolic Risk: Association With Genetic, Biological, and Environmental Factors. J Diabetes Res(2015) (2015) 783296. doi: 10.1155/2015/783296 PMC4530255 26273675

[45] Gätjens I Schmidt SCE Plachta-Danielzik S Bosy-Westphal A Müller MJ . Body Composition Characteristics of a Load-Capacity Model: Age-Dependent and Sex-Specific Percentiles in 5- to 17-Year-Old Children. Obes Facts (2021) 14(6):593–603. doi: 10.1159/000518638 34818246 PMC8738913

[46] Mueske NM Mittelman SD Wren TAL Gilsanz V Orgel E . Myosteatosis in Adolescents and Young Adults Treated for Acute Lymphoblastic Leukemia. Leuk Lymphoma (2019) 60(13):3146–53. doi: 10.1080/10428194.2019.1623889 PMC6923569 31264493

[47] Yodoshi T Orkin S Arce Clachar AC Bramlage K Sun Q Fei L . Muscle Mass Is Linked to Liver Disease Severity in Pediatric Nonalcoholic Fatty Liver Disease. J Pediatr (2020) 223:93–99.e2. doi: 10.1016/j.jpeds.2020.04.046 32711755 PMC8017767

[48] Stefanaki C Peppa M Boschiero D Chrousos GP . Healthy Overweight/Obese Youth: Early Osteosarcopenic Obesity Features. Eur J Clin Invest (2016) 46(9):767–78. doi: 10.1111/eci.12659 27434725

[49] Joffe L Shen W Shadid G Jin Z Ladas EJ . Skeletal Muscle and Adipose Tissue Changes in the First Phase of Treatment of Pediatric Solid Tumors. Cancer Med (2021) 10(1):15–22. doi: 10.1002/cam4.3584 33140912 PMC7826460

[50] Moher D Liberati A Tetzlaff J Altman DG . PRISMA Group. Preferred reporting items for systematic reviews and meta-analyses: the PRISMA statement. PLoS Med (2009) 6(7):e1000097 10.1371/journal.pmed.1000097 PMC2707599 19621072

[51] Lim S Kim JH Yoon JW Kang SM Choi SH Park YJ . Sarcopenic Obesity: Prevalence and Association With Metabolic Syndrome in the Korean Longitudinal Study on Health and Aging (KLoSHA). Diabetes Care (2010) 33(7):1652–4. doi: 10.2337/dc10-0107 PMC2890376 20460442

[52] Chung JY Kang HT Lee DC Lee HR Lee YJ . Body Composition and its Association With Cardiometabolic Risk Factors in the Elderly: A Focus on Sarcopenic Obesity. Arch Gerontol Geriatr (2013) 56:270–8. doi: 10.1016/j.archger.2012.09.007 23079031

[53] Kim TN Park MS Kim YJ Lee EJ Kim MK Kim JM . Association of Low Muscle Mass and Combined Low Muscle Mass and Visceral Obesity With Low Cardiorespiratory Fitness. PloS One (2014) 9(6):e100118. doi: 10.1371/journal.pone.0100118 24937121 PMC4061126

[54] Hong N Lee EY Kim CO . Gamma-Glutamyl Transferase is Associated With Sarcopenia and Sarcopenic Obesity in Community-Dwelling Older Adults: Results From the Fifth Korea National Health and Nutrition Examination Survey, 2010–2011. Endocr J (2015) 62:585–92. doi: 10.1507/endocrj.EJ15-0119 25913781

[55] Carias S Castellanos AL Vilchez V Nair R Dela Cruz AC Watkins J . Nonalcoholic Steatohepatitis is Strongly Associated With Sarcopenic Obesity in Patients With Cirrhosis Undergoing Liver Transplant Evaluation. J Gastroenterol Hepatol (2016) 31(3):628–33. doi: 10.1111/jgh.13166 PMC6615558 26399838

[56] Schrager MA Metter EJ Simonsick E Ble A Bandinelli S Lauretani F . Sarcopenic Obesity and Inflammation in the InCHIANTI Study. J Appl Physiol (1985) (2007) 102(3):919–25. doi: 10.1152/japplphysiol.00627.2006 PMC2645665 17095641

[57] Hamer M Batty GD Kivimaki M . Sarcopenic obesity and risk of new onset depressive symptoms in older adults: English Longitudinal Study of Ageing. Int J Obes (Lond) (2015) 39(12):1717–20. doi: 10.1038/ijo.2015.124 PMC4722238 26122029

