# Corrigendum: Sarcopenic obesity in children and adolescents: a systematic review

**DOI:** 10.3389/fendo.2023.1269546

**Published:** 2023-08-15

**Authors:** Marcela Zembura, Paweł Matusik

**Affiliations:** Department of Pediatrics, Pediatric Obesity and Metabolic Bone Diseases, Chair of Pediatrics and Pediatric Endocrinology, Faculty of Medical Sciences in Katowice, Medical University of Silesia, Katowice, Poland

**Keywords:** sarcopenia, obesity, muscle mass, muscle strength, children, adolescents

## Incorrect Reference

In the published article, the reference 12 was incorrectly written as “NIH.Study Quality Assessment Tools. Available at: https://www.nhlbi.nih.gov/health-topics/study-quality-assessment-tools (Accessed 1.12.2021)”. The correct reference 12 should be “Woo J. Sarcopenia. Clin Geriatr Med (2017) *33*(3), 305–314. https://doi.org/10.1016/j.cger.2017.02.003.”

The authors apologize for this error and state that this does not change the scientific conclusions of the article in any way. The original article has been updated.

## Text Corrections

In the published article, there was an error in the first sentence of the section **3 Results**, *3.1 Sarcopenic Obesity Evaluation Methods*. This sentence previously stated:

“*Overall* DXA (n = 8/18) (33, 35, 37, 38, 41, 43, 44, 46) and analysis BIA (n = 6/18) (25, 34, 36, 42, 45, 48) were the most commonly used body composition evaluation methods, followed by other imaging techniques (4/18) (40, 46, 47, 49) and assessment of handgrip strength (HGS) using dynamometer (39).”

The corrected sentence appears below:

“*Overall* DXA (n = 8/18) (33, 35, 37, 38, 41, 43, 44, 46) and BIA (n = 6/18) (25, 34, 36, 42, 45, 48) were the most commonly used body composition evaluation methods, followed by other *imaging* techniques (4/18) (40, 46, 47, 49) and assessment of handgrip strength (HGS) using dynamometer (39).”

The authors apologize for this error and state that this does not change the scientific conclusions of the article in any way. The original article has been updated.

In the published article, there was an error in the first sentence of the section **5 Conclusions**.

This sentence previously stated:

“In conclusion, in our review, the prevalence of SO ranged from 984 5.66% to 69.7% in girls, with a range between 7.2% and 81.3% in 985 boys SO.”

The corrected sentence appears below:

“In conclusion, in our review, the prevalence of SO ranged from 5.66% to 69.7% in girls, with a range between 7.2% and 81.3% in boys.”

The authors apologize for this error and state that this does not change the scientific conclusions of the article in any way. The original article has been updated.

